# Draft Genome Sequence of Propane- and Butane-Oxidizing Actinobacterium *Rhodococcus ruber* IEGM 231

**DOI:** 10.1128/genomeA.01297-14

**Published:** 2014-12-11

**Authors:** Irena B. Ivshina, Maria S. Kuyukina, Anastasiya V. Krivoruchko, Valérie Barbe, Cécile Fischer

**Affiliations:** a Institute of Ecology and Genetics of Microorganisms, Ural Branch, Russian Academy of Sciences, Perm, Russia; b Perm State University, Perm, Russia; c CEA/DSV/IG/Genoscope/LF, Évry, France; d Université Val d’Essonne–UMR8030 and CEA/DSV/IG/Laboratoire de Génomique et Biochimie du Métabolisme and CNRS, Évry, France

## Abstract

We report a draft genome sequence of *Rhodococcus ruber* IEGM 231, isolated from a water spring near an oil-extracting enterprise (Perm region, Russian Federation). This sequence provides important insights into the genetic mechanisms of propane and *n*-butane metabolism, organic sulfide and beta-sitosterol biotransformation, glycolipid biosurfactant production, and heavy metal resistance in actinobacteria.

## GENOME ANNOUNCEMENT

Nocardioform actinobacteria of the species *Rhodococcus ruber* grow on the gaseous hydrocarbons ethane, propane, and *n-*butane as sole carbon and energy sources and can be used in prospecting for gas and oil deposits, as well as in other technologies (1–5). The *R. ruber* strain IEGM 231 was isolated from a water spring near an oil-extracting enterprise (Perm region, Russian Federation) and deposited in the IEGM Collection of Alkanotrophic Microorganisms (acronym IEGM, no. 768 WDCM, http://www.iegm.ru/iegmcol/strains/rhodoc/ruber/r_ruber231.html) (6). The strain was shown in our laboratory to utilize propane, *n*-butane, and liquid alkanes (C_12_–C_17_); convert aryl alkyl sulfides into optically pure sulfoxides; transform beta-sitosterol into therapeutically prospective stigmast-4-en-3-one; produce nontoxic glycolipid biosurfactants; have a high resistance to heavy metals; and accumulate molybdenum and nickel.

The whole genome of *Rhodococcus ruber* IEGM 231 was sequenced using Illumina technology. A mate-paired library with an insert size of 6 kb was produced and sequenced with the MiSeq system (2 × 250 nt). An approximately 130-fold coverage was generated, and the data were assembled with the Velvet assembler (https://www.ebi.ac.uk/~zerbino/velvet). Gap filling was performed using GapCloser (http://soap.genomics.org.cn/soapdenovo.html) on scaffolds more than 2 kb in size. The annotation of coding sequences (CDS) and prediction of gene functions were performed using the MicroScope platform (https://www.genoscope.cns.fr/agc/microscope/home/index.php) (7), which integrated 115 contigs, resulting in 46 scaffolds from the assembly. The genome size of *R. ruber* IEGM 231 is estimated to be 6.01 Mb with a G+C content of 70.22%.

A total of 5,928 CDSs with an average length of 948 bp, 6 rRNAs, and 53 tRNAs were found in the *R. ruber* IEGM 231 genome. At least 73 CDSs coded for monooxygenases/hydroxylases, 22 CDSs coded for cytochromes P450, 45 CDSs coded for dioxygenases, 13 CDSs coded for peroxidases, and 285 CDSs coded for dehydrogenases. Among monooxygenases, genes coding for 2 putative propane monooxygenases *mmoABC* and *prmA*, 2 alkane 1-monooxygenases *alkB*, 9 flavin monooxygenases, and 1 cyclohexanone 1,2-monooxygenase were revealed, reinforcing the strong phenotypic abilities of the species toward hydrocarbons. Diversity of these sequences could also be evidence of the existence of several alkane and organic sulfide degradation systems in *R. ruber* IEGM 231 (8, 9). Two *choD* (coding for cholesterol oxidase) homologues and 1 gene homologous to 3-beta hydroxysteroid dehydrogenase/isomerase were present. They could account for steroid compound transformations, as already shown for other *R. ruber* strains (10, 11). The diversity of CDSs coding for glycolipid biosurfactant synthesis was presented by 1 malonyl CoA-ACP transacylase, 14 acyl-CoA synthetases, 1 fatty acid synthase I, 9 3-oxoacyl-ACP reductases, 1 cyclopropane mycolic acid synthase, 1 polyketide synthase, 1 meromycolate extension ACP, 3 mycolyltransferases, 1 maltooligosyl trehalose synthase, 1 maltooligosyl trehalose trehalohydrolase, and 1 trehalose synthase. Among heavy metal–associated CDSs, there were CDSs coding for heavy metal resistance proteins (5 CDSs), heavy metal transporters (8 CDSs), cation efflux enzymes (3 CDSs), metal binding proteins (3 CDSs), a mercuric reductase, and an alkylmercuryl lyase.

### Nucleotide sequence accession numbers.

This whole-genome shotgun project has been deposited at DDBJ/EMBL/GenBank under accession numbers CCSD01000001 to CCSD01000115.
